# Circadian coordination of ATP release in the urothelium via connexin43 hemichannels

**DOI:** 10.1038/s41598-018-20379-0

**Published:** 2018-01-31

**Authors:** Atsushi Sengiku, Masakatsu Ueda, Jin Kono, Takeshi Sano, Nobuyuki Nishikawa, Sumihiro Kunisue, Kojiro Tsujihana, Louis S. Liou, Akihiro Kanematsu, Shigeki Shimba, Masao Doi, Hitoshi Okamura, Osamu Ogawa, Hiromitsu Negoro

**Affiliations:** 1 Department of Urology, Graduate School of Medicine, Kyoto University, Kyoto, 606-8507 Japan; 2 Department of Urology, Japanese Red Cross Otsu Hospital, Shiga, 520-8511 Japan; 3 Department of Systems Biology, Graduate School of Pharmaceutical Sciences, Kyoto University, Kyoto, 606-8501 Japan; 4 Department of Dermatology, Graduate School of Medicine, Kyoto University, Kyoto, 606-8507 Japan; 5 Department of Urology, Cambridge Health Alliance, Cambridge, MA 02139 USA; 6 Department of Urology, Hyogo College of Medicine, Hyogo, 663-8501 Japan; 7 Department of Health Science, School of Pharmacy, Nihon University, Chiba, 245-8555 Japan

## Abstract

Day-night changes in the storage capacity of the urinary bladder are indispensable for sound sleep. Connexin 43 (Cx43), a major gap junction protein, forms hemichannels as a pathway of ATP in other cell types, and the urinary bladder utilizes ATP as a mechanotransduction signals to modulate its capacity. Here, we demonstrate that the circadian clock of the urothelium regulates diurnal ATP release through Cx43 hemichannels. Cx43 was expressed in human and mouse urothelium, and clock genes oscillated in the mouse urothelium accompanied by daily cycles in the expression of Cx43 and extracellular ATP release into the bladder lumen. Equivalent chronological changes in Cx43 and ATP were observed in immortalized human urothelial cells, but these diurnal changes were lost in both arrhythmic *Bmal1*-knockout mice and in *BMAL1-*knockdown urothelial cells. ATP release was increased by *Cx43* overexpression and was decreased in *Cx43* knockdown or in the presence of a selective Cx43 hemichannel blocker, which indicated that Cx43 hemichannels are considered part of the components regulating ATP release in the urothelium. Thus, a functional circadian rhythm exists in the urothelium, and coordinates Cx43 expression and function as hemichannels that provide a direct pathway of ATP release for mechanotransduction and signalling in the urothelium.

## Introduction

Humans have the ability to increase bladder capacity and decrease urine production at night-time compared with daytime to avoid disturbances of sleep by micturition^1–3^. Nocturnal enuresis in childhood and frequent nocturia in the elderly are common and are detrimental to the quality of life by interfering with the patients’ sleeping habits^4–8^. Insufficient bladder capacity, excessive urine production or both at night-time are the main causes of nocturnal enuresis or frequent nocturia^1,9,10^. Therefore, clarification of the mechanism(s) of nocturnal enlargement of the bladder would help improve these clinical complaints.

Previously, we have reported that the circadian clock regulated connexin 43 (Cx43), a major gap junction protein, in mouse/rat bladder muscle cells^11^. Mice are nocturnal (i.e. active primarily during the night), and the phases of their sleep-wake cycles are inverted to those of humans. Their bladder capacity is increased during the sleep/light phase and decreased during the active/dark phase. Cx43 levels in the bladder and the functional bladder capacity showed consistent circadian oscillations in wild-type mice, and the expression level of Cx43 protein in the rat/mouse bladder was inversely correlated with functional bladder capacity. The functional bladder capacity was higher in heterozygous *Cx43*^+/−^ mice than that in *Cx43*^+/+^ mice. However, the circadian rhythms of Cx43 expression level and functional bladder capacity were completely lost in *Cry*-null mice, which have a dysfunctional circadian clock^11^. The clock regulator, Rev-erbα, upregulated *Cx43* transcription as a co-factor of Sp-1 using Sp-1 cis-elements of the promoter^12^. However, our previous report focused on bladder muscle cells^11–14^, and the roles of other components that determine bladder capacity are still unclear.

The urothelium has prominent morphological changes, termed transitional epithelium. The urothelium is a stratified epithelium composed of several layers — the superficial, intermediate, and basal cell layers — that change their morphology in relation to the filling state of the bladder^15,16^. Urothelial cells appear to be cuboidal when the urinary bladder is not stretched in the vacant phase, and the cells become stretched to a flattened shape when the bladder is filled with urine. Thus, urothelial cells stretch to accommodate increase in urine volume. The primary function of the urothelium is a barrier to prevent the entry of pathogens into the underlying tissues. To achieve this, urothelial cells are connected by tight junctions, or virtually impenetrable junctions that seal the cellular membranes of neighbouring cells^17,18^.

Urothelial cells also sense changes in stretch and/or intravesical pressure and transmit mechanotransduction signals to the afferent nerve by releasing various neurotransmitters^19,20^. Of them, ATP released to the basal side of the urothelium activates purinergic receptors such as P2X_2_ and P2X_3_ expressed by afferent nerves in the suburothelium^21–25^, whereas ATP released to the luminal side activates various P2X and P2Y receptors on the urothelium in an autocrine/paracrine manner to form a positive feedback loop of ATP secretion^26,27^. These findings suggest that ATP modulates bladder capacity by transmitting mechanotransduction signals.

In general, there are two pathways for cellular ATP release — vesicular and non-vesicular mechanisms. Vesicular ATP release involves exocytosis^28^ while non-vesicular ATP release might be mediated by mechanical stretch, activation of voltage and/or ligand-gated ion channels and receptors, mitochondrial porins, ATP binding cassette transporters, *Pannexin1 (PANX1)*^27,29^, *Transient Receptor Potential Vanilloid4 (TRPV4)*^30^ and *PIEZO1*^31^. Recently, it has been reported that Cx43 forms hemichannels in the pathway for non-vesicular ATP release in bone cells and astrocytes^32,33^.

Based on the above, we hypothesized that the increased functional bladder capacity during sleep-time at night was promoted by the decrease of mechanotransduction signalling by ATP through C43 hemichannels in the urothelium via the functional circadian clock. Here, we investigated the circadian role of the urothelium for ATP release through regulating Cx43 expression and the function of hemichannels in urothelial cells in mice and humans.

## Results

### Cx43 is expressed in human and mouse urothelium

**Figure 1:**
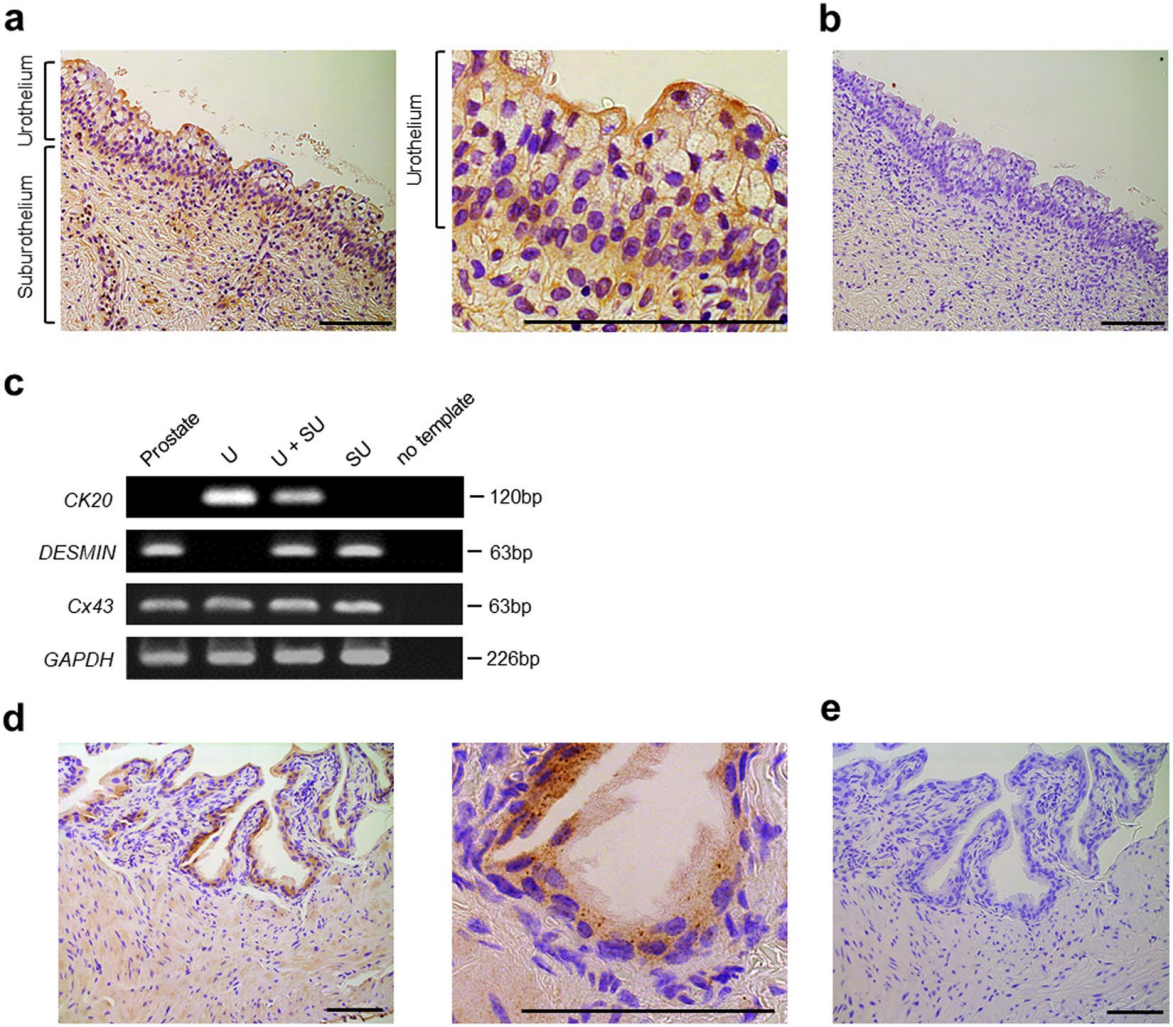
Cx43 immunohistochemistry (IHC) in normal human/mouse bladder mucosa. (**a**) The normal human urothelium shows positive Cx43 staining. (**b**) IHC with only the secondary antibody as a negative control. (**c**) Normal human urothelium expresses Cx43 in the urothelium (U) and the suburothelium (SU) by RT-PCR analysis. Cytokeratin 20 (CK20) and desmin were used as urothelial and suburothelial markers, respectively. Normal human prostate was used as a reference. (**d**) Normal mouse urothelium shows positive Cx43 staining. (**e**) IHC with only the secondary antibody was used as a negative control. All scale bars indicate 100 µm.

We determined whether Cx43 is expressed in the human urothelium by immunostaining and reverse transcriptional polymerase chain reaction (RT-PCR) of *Cx43* in non-pathological human bladder tissue. The normal human urothelium showed positive staining for Cx43 (Fig. 1a,b). Within the urothelium, basal cells strongly expressed Cx43 as reported in rats^34^, and umbrella and intermediate cells mildly expressed Cx43 with a scattered staining pattern. RT-PCR demonstrated the expression of *Cx43* in the urothelium (Fig. 1c). These results confirmed normal human urothelium contains Cx43 expression.

We also examined whether Cx43 is expressed in the mouse urothelium. We performed immunostaining and detected Cx43 expression in normal mouse urothelium (Fig. 1d,e). In addition, we conducted layer-specific dissection (Supplementary Fig. S1a) and found considerable *Cx43* mRNA expression in the urothelium by real-time quantitative RT-PCR analysis. Immunoblot analysis with layer-specific samples further confirmed the presence of Cx43 in the urothelium (Supplementary Fig. S1b). These findings demonstrate that Cx43 is expressed in human and mouse urothelium.

### Mice urothelium contains an endogenous circadian clock and Cx43 oscillation

**Figure 2:**
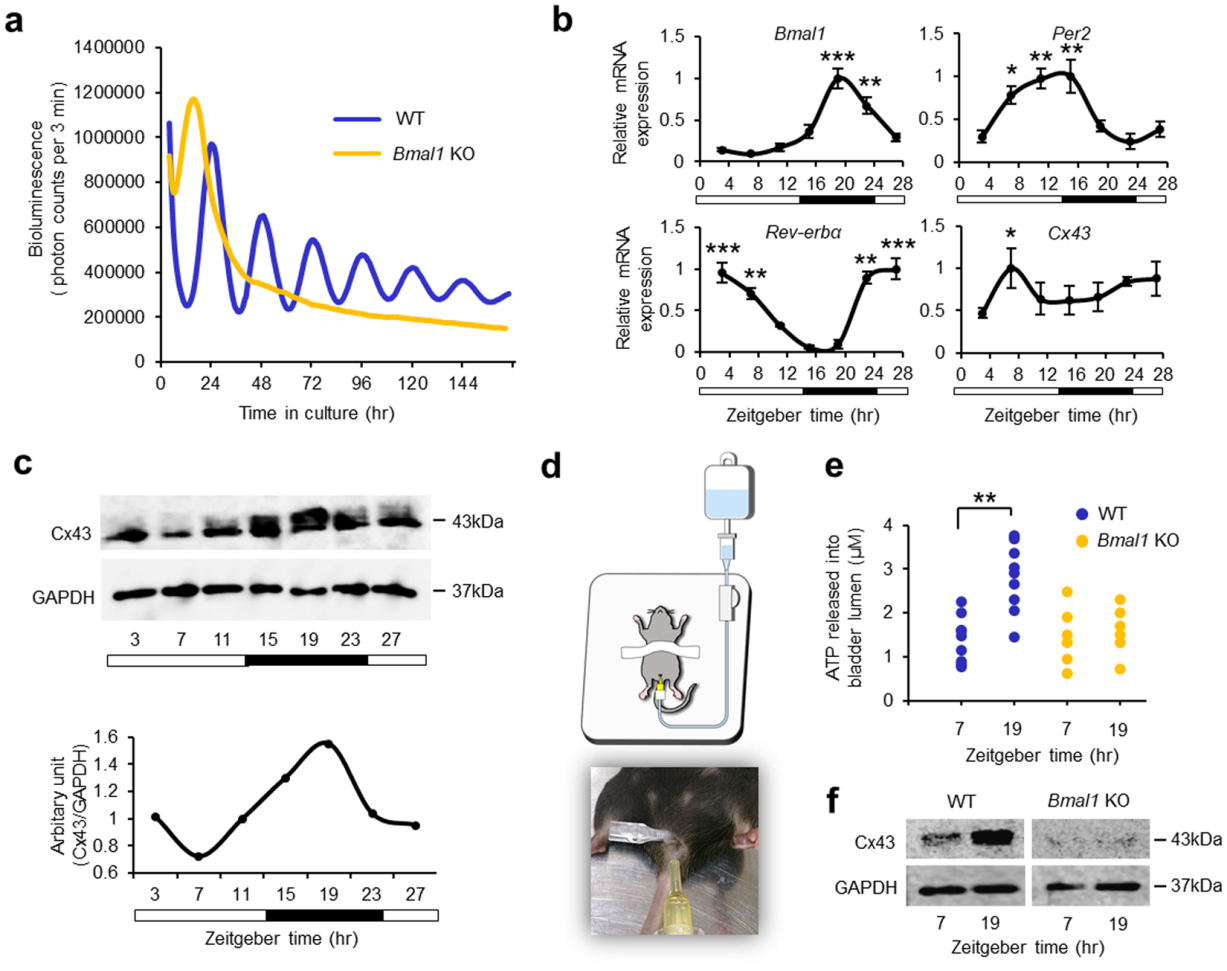
Oscillation of Cx43 and Clock gene expression in mouse urothelium. (**a**) Representative oscillation of bioluminescence in the urothelium obtained from *Per2::luc* mice from an *ex vivo* culture. The period of oscillation was 24.13 ± 0.07 (mean ± s.e.m) (n = 3). The oscillation of bioluminescence in *Per2::luc/Bmal1*^−/−^ mice were lost completely. (**b**) Temporal mRNA accumulation of *Bmal1*, *Per2*, *Rev-erbα* and *Cx43* in the urothelium from mice under LD condition. *P < 0.05, **P < 0.01 and ***P < 0.001 vs. the nadir value (ZT 7 in *Bmal1*, ZT 23 in *Per2*, ZT 15 in *Rev-erbα* and ZT 3 in *Cx43*) by one-way ANOVA with Tukey’s *post hoc* test (n = 4 for each time point). The *P*-values with Cosinor analysis in *Bmal1*, *Per2*, *Rev-erbα and Cx43 were* 0.02, 0.002, 0.005 and 0.1 respectively. ZT; Zeitgeber time. (**c**) Representative temporal Cx43 protein accumulation in the mice urothelium under LD condition as shown by immunoblotting (Cosinor analysis, *P* = 0.01). Full-length blots are presented in Supplementary Fig. S11. (**d**) A schematic image and photograph of bladder distention to measure the concentration of ATP released into the bladder. (**e**) Temporal change in the concentration of ATP release after bladder distention. The high/low level was in accord with the Cx43 expression in the urothelium of mice. **P < 0.01 by Student’s *t*-test (n = 9), which was completely lost in *Bmal1*-knockout mice (n = 6). (**f**) Circadian change of Cx43 protein expression in the urothelium of wild-type mice was lost in *Bmal1*-knockout mice. Full-length blots are presented in Supplementary Fig. S11. All error bars indicate s.e.m.

Because the distribution and expression of Cx43 are similar in humans and mice, we used mouse urothelium to characterize the circadian role of Cx43 in urothelial cells. To examine whether mouse urothelium *per se* has a circadian clock, we characterized the endogenous clock oscillation in *ex vivo* cultured urothelium isolated from *Per2*^*Luciferase*^ knock-in (*Per2::luc*) gene reporter mice. We found a robust *Per2::luc* oscillation of at least six cycles (Fig. 2a, Supplementary Fig. S2) with a period of oscillation of 24.13 ± 0.07 (mean ± s.e.m.). This oscillation was not observed in *Per2::luc Bmal1*-knockout mice (*Per2::luc/Bmal1*^−/−^) (Fig. 2a). These results demonstrate that the bladder urothelium has an endogenous oscillatory machinery.

However, it was unknown whether these cellular oscillations of the circadian clock persisted in the bladder urothelium *in vivo*. We collected urothelium from mouse bladders at seven consecutive time points every 4 hours during the day (zeitgeber time (ZT) 0, 4, 8, 12, 16, 20 and 24) to elucidate whether the circadian clocks exist in the urothelium. RT-PCR analysis demonstrated that the major clock genes *Bmal1*, *Per2* and *Rev-erbα* mRNA had robust diurnal rhythms, that *Cx43* mRNA also had diurnal variation in the urothelium (Fig. 2b), and that Cx43 protein was cycled in abundance with a diurnal rhythm (Fig. 2c). Immunoblot analysis demonstrated Cx43 peaked at the middle of the active/dark phase for mice, but was lowest at the middle of the sleep/light phase. The peak of Cx43 protein expression was delayed for 8–12 hrs from the peak of *Cx43* mRNA expression. These results suggested that a post-transcriptional mechanism was required to induce the circadian rhythm of Cx43 protein expression from the diurnal variation of mRNA expression. These findings demonstrate that the levels of Cx43 display diurnal rhythms in the mouse urothelium *in vivo*, in conjunction with its circadian regulator, *Rev-erbα*, as we reported in the bladder smooth muscle^11^.

### ATP released into the bladder lumen undergoes daily variations in mice

We next examined whether ATP released from the urothelium had a diurnal variation in mice. We measured the concentration of ATP in the bladder lumen after bladder distention with 30 cm H_2_O for 10 minutes at ZT7, a resting phase for mice, and ZT19, an active phase (Fig. 2d). The concentration of ATP was significantly higher at ZT19 than at ZT7 (p < 0.01), which was in accord with Cx43 expression in the urothelium in wild-type mice. However, such diurnal changes in ATP release and Cx43 protein expression was lost in arrhythmic *Bmal1*-knockout mice (Fig. 2e,f). These results suggest that ATP release from the urothelium induced by bladder distension undergoes daily variations regulated by the circadian clock and that changes in ATP release in the resting phase versus the active phase may be involved in the daily oscillation of functional bladder capacity.

### Immortalized human urothelial cells exhibit circadian cycles of Cx43 expression and ATP release

We found circadian profiles of the clock genes, as well as Cx43 and ATP release in the urothelium. To investigate the causal-relationship among these findings, we used hTERT-immortalized human urothelial cells (TRT-HU1)^27,35–37^.

**Figure 3:**
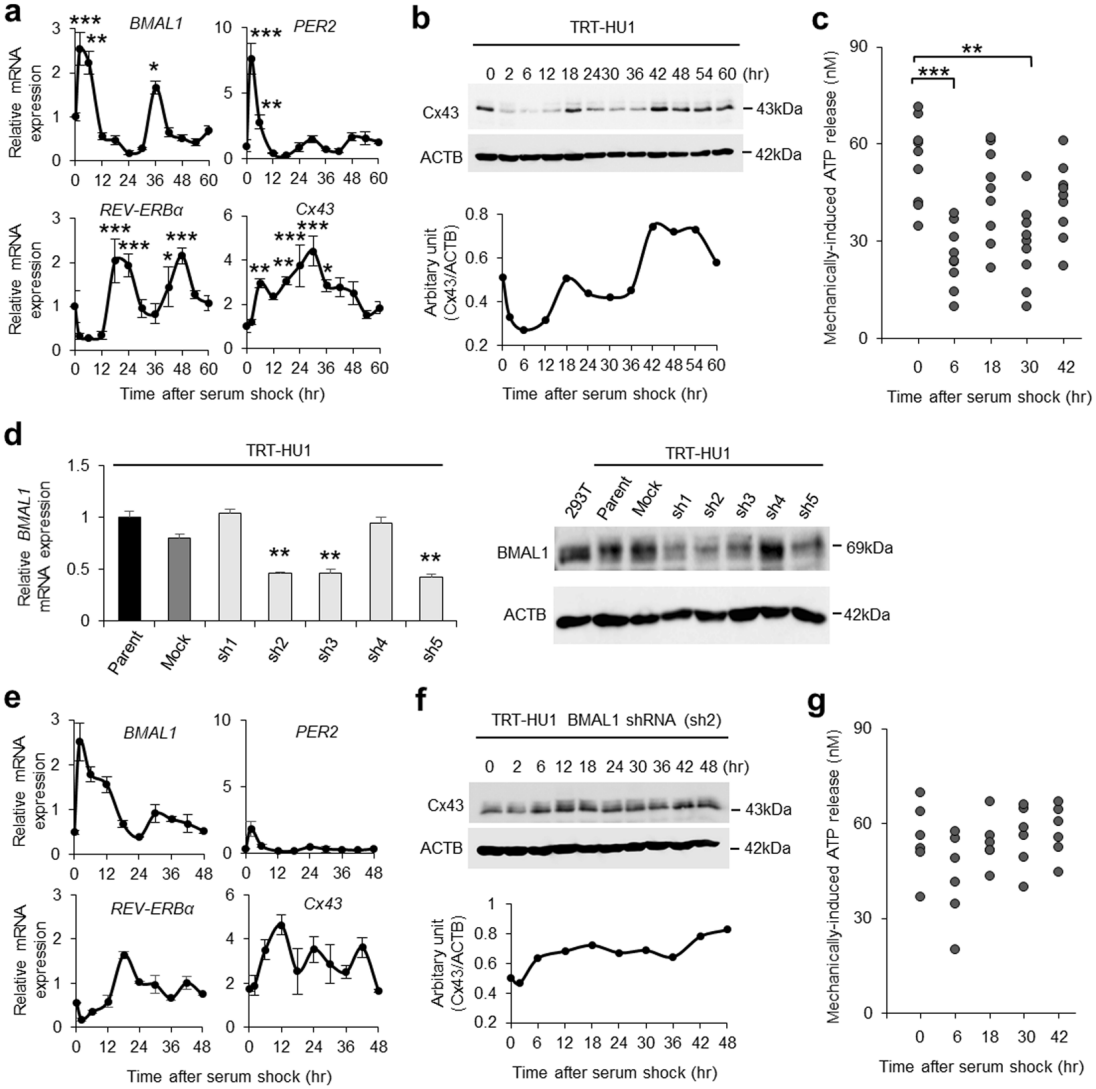
Oscillation of Cx43 and Clock gene expression in immortalized human urothelial cells. (**a**) Temporal variation of mRNA accumulation of clock genes and *Cx43* mRNA levels in serum-shocked immortalized human urothelial cells (TRT-HU1). The major clock genes *BMAL1*, *PER2*, and *REV-ERBα* had circadian rhythms.*P < 0.05, **P < 0.01 and ***P < 0.001 vs. the nadir value (time 24 in *BMAL1*, time 18 in *PER2*, time 6 in *REV-ERBα* and time 0 in *Cx43*) by one-way ANOVA with Dunnett’s *post hoc* test (n = 3). The *P*-values with Cosinor analysis in *Bmal1*, *Per2*, *Rev-erbα and Cx43 were* 0.01, 0.0001, 0.002 and 0.09, respectively. (**b**) Representative immunoblots showing temporal changes in Cx43 protein levels in serum-shocked TRT-HU1 (Cosinor analysis, *P* = 0.006). Full-length blots are presented in Supplementary Fig. S12. (**c**) Temporal variation of mechanically induced ATP release in TRT-HU1 after serum-shock. **P < 0.01 and *** *P* < 0.001 by one-way ANOVA with Dunnett’s *post hoc* test (n = 9). (**d**) Stable knockdown of *BMAL1* in TRT-HU1 (sh1-sh5). **P < 0.01 vs. mock by Student’s *t*-test (n = 3). Full-length blots are presented in Supplementary Fig. S12. (**e**) Temporal variation of mRNA accumulation of clock genes and *Cx43* mRNA levels in serum-shocked sh2 TRT-HU1 (TRT-HU1 *BMAL1* shRNA). (**f**) Representative immunoblots showing disturbed temporal changes in Cx43 protein levels in serum-shocked *BMAL1* shRNA TRT-HU1. Full-length blots are presented in Supplementary Fig. S12. (**g**) Disturbed temporal variation of mechanically induced ATP release after serum-shocked sh2 TRT-HU1 (n = 6). All error bars indicate s.e.m.

Before examining these relationships, we investigated whether this cell line was suitable for circadian study. After synchronization of the circadian clock by serum shock after applying 50% FBS^38^, we assessed Cx43 protein and mRNA levels every 6 hours in individual groups for 60 hours. The major clock genes *BMAL1*, *PER2*, and *REV-ERBα* mRNA had circadian rhythms and *Cx43* mRNA showed a variation *in vitro* similar to the urothelium of mice *in vivo* (Fig. 3a). In addition, Cx43 protein showed an overt circadian rhythm in immunoblot analysis (Fig. 3b). ATP release induced by rinsing with cell bathing solution showed a significantly lower concentration at 6 and 30 hours after serum shock compared with control at 0 hour before serum shock (Fig. 3c). This variation in ATP release coincided with the change of Cx43 protein expression in the human urothelial cells (Fig. 3b). The synchronous circadian oscillations of Cx43 expression and ATP release suggest a functional correlation between Cx43 and ATP.

To demonstrate that the Cx43 expression and ATP release are under the control of the circadian clock, we investigated the influence of *BMAL1*-knockdown on the circadian rhythm in urothelial cells. After the transfection of five different *BMAL1* shRNA plasmids (sh1-5) to TRT-HU1, *BMAL1* shRNA plasmid (sh2) transfected cells (sh2 TRT-HU1) had the highest efficacy in *BMAL1* knockdown by real-time quantitative RT-PCR and immunoblotting (Fig. 3d). After serum shock, the oscillation of *PER2* and *REV-ERBα* as well as *BMAL1* mRNA expression became unclear in sh2 TRT-HU1 compared with that in parent TRT-HU1 (Fig. 3e, Supplementary Fig. S3). Accordingly, the oscillation of Cx43 protein expression was lost in sh2 TRT-HU1 (Fig. 3f). In addition, the circadian rhythm of mechanically induced ATP release disappeared in sh2 TRT-HU1 (Fig. 3g). Furthermore, to confirm whether *Cx43* mRNA expression was regulated by Rev-erbα and Sp-1 in urothelial cells as in smooth muscle cells^11^, a *Cx43* promoter-reporter assay was performed using TRT-HU1. As a result, *Cx43* transcription was upregulated in coexistence with Rev-erbα and Sp-1 in TRT-HU1 (Supplementary Fig. S4). These results suggest that the circadian clock system may regulate the oscillation of Cx43 expression and ATP release in urothelial cells.

### ATP is released from Cx43 hemichannels in urothelial cells

**Figure 4:**
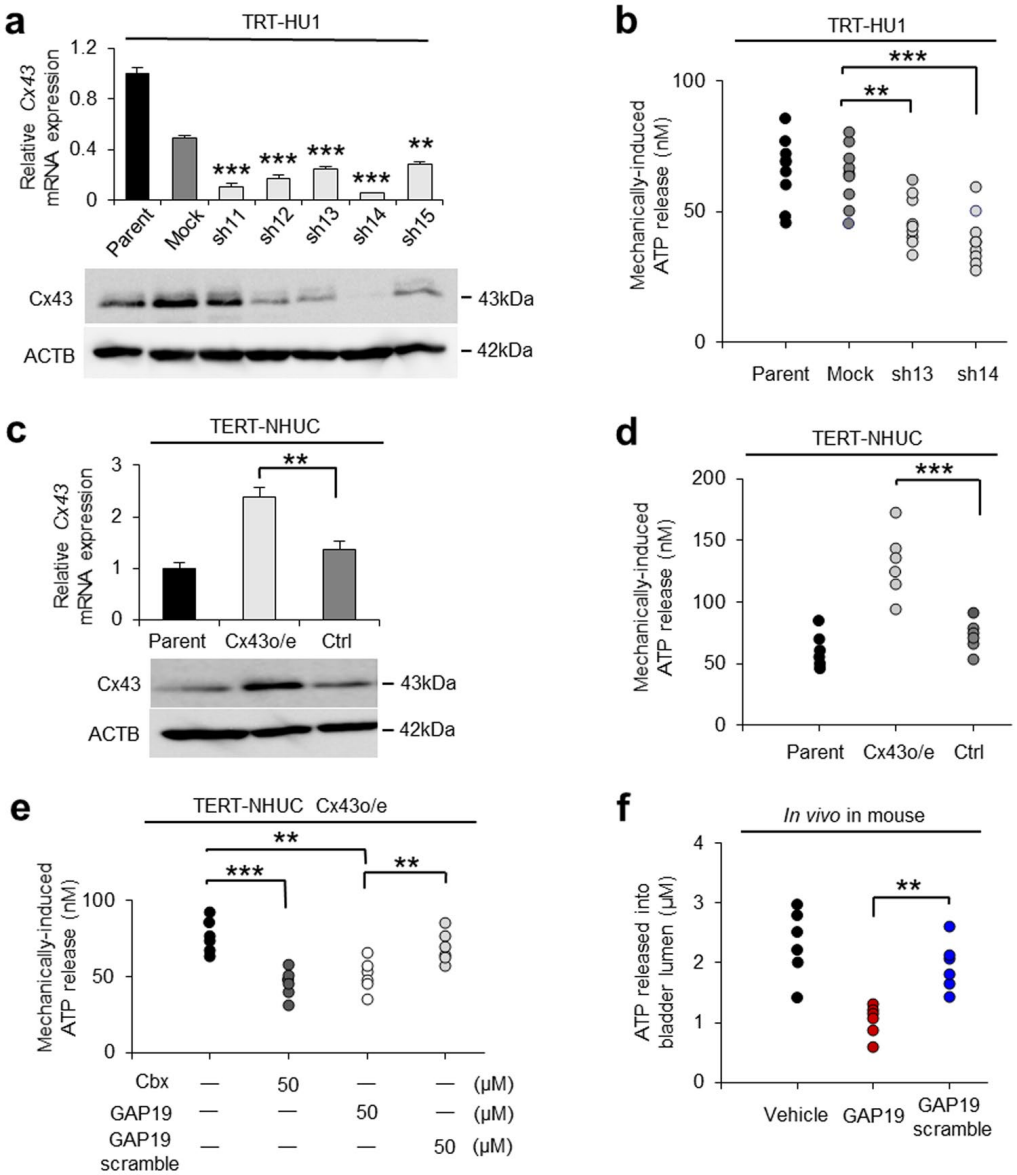
Mechanically induced ATP release via Cx43 hemichannels has diurnal variation. (**a**) Stable knockdown of *Cx43* in TRT-HU1 (sh11-sh15). **P < 0.01 and ***P < 0.001 vs. mock by Student’s *t*-test (n = 3). Full-length blots are presented in Supplementary Fig. S13. (**b**) Mechanically-induced ATP release was decreased in both sh13 and sh14 TRT-HU1. **P < 0.01 and ***P < 0.001 by Student’s *t*-test (n = 9). (**c**) *Cx43*-overexpression in TERT-NHUC (*Cx43* o/e TERT-NHUC). **P < 0.01 by Student’s *t*-test (n = 3). Full-length blots are presented in Supplementary Fig. S13. (**d**) Increased mechanically induced ATP release in *Cx43* o/e TERT-NHUC. ***P < 0.001 by Student’s *t*-test (n = 6). (**e**) Mechanically-induced ATP release in the presence of carbenoxolone and GAP19 peptide. Each reagent was used at 50 µM. **P < 0.001 by Student *t*-test (n = 6). (**f**) The concentration of ATP release after bladder distention in the presence of GAP19 peptide and GAP19 scramble peptide *in vivo* in mice at ZT19. **P < 0.01 by Student’s *t*-test (n = 6). All error bars indicate s.e.m.

To investigate whether Cx43 is one of the pathways of ATP release in urothelial cells, we generated *Cx43* knockdown and overexpressing immortalized human urothelial cells by using two types of immortalized human urothelial cells — TRT-HU1 and TERT-NHUC^39,40^. As for the *BMAL1*-knockdown procedure, the transfection of five different *Cx43* shRNA plasmids (sh11-15) to TRT-HU1 indicated that the shRNA14 (sh14 TRT-HU1) and sh13 (sh13 TRT-HU1) plasmids had the two highest efficacies (Fig. 4a, Supplementary Fig. S5). The concentration of mechanically induced ATP release was significantly lower in sh13 and sh14 TRT-HU1 cells compared with that in control cells (Fig. 4b). However, *Cx43*-overexpressing TERT-NHUC showed a significantly higher concentration of mechanically induced ATP release compared with the control cells (Fig. 4c,d, Supplementary Fig. S6). In these two *Cx43* knockdown/overexpressing cell types, mRNA expressions of *BMAL1* and other candidate genes associated with urothelial ATP release, *PANX1*^26,27^, *TRPV4*^30^, *PIEZO1*^31^ and *Vesicular Nucleotide Transporter (VNUT)*^28^ were not correlated with the increase/decrease of ATP release (Supplementary Figs S7, S8). These results suggest that ATP release from urothelial cells is under the direct control of *Cx43* rather than that of *Bmal1* or other potential genes for the activation of ATP release driven by *Bmal1* that are yet to be identified.

In addition, mechanically induced ATP release was significantly reduced in the presence of carbenoxolone (Cbx), a gap junction and hemichannel inhibitor, compared with the control cells. To clarify the role of the two types of Cx43 channels, we used GAP19 peptide^41,42^, a specific Cx43 hemichannel blocker. Mechanically induced ATP release was significantly lower in the presence of the GAP19 peptide than that in the presence of scramble control of GAP19 peptide (Fig. 4e), while dye-transfer length indicating gap junction function was not prevented in the GAP19 peptide (Supplementary Fig. S9a) different from using sh14 TRT-HU1 cells that significantly decreased the length (Supplementary Fig. S9b). These results suggest that urothelial cells employ Cx43 hemichannel rather than Cx43 gap-junction for ATP release.

To investigate whether Cx43 hemichannels function *in vivo*, GAP19 peptide was used for the examination of ATP release by bladder distention in mice at ZT19. The concentration of ATP release was significantly lower in the presence of GAP19 peptide than that in the presence of GAP19 scramble peptide (Fig. 4f). Based on these results, Cx43 hemichannels are considered part of the components regulating ATP release in the urothelium. Therefore, we propose that circadian rhythm in the urothelium coordinates the diurnal change in ATP release via Cx43 hemichannels, which modulate functional bladder capacity (Supplementary Fig. S10).

## Discussion

The fundamental adaptive advantage of the circadian clock is that it allows for predictive, rather than entirely reactive, homeostatic regulation of physiological functions. Diurnal change in functional bladder capacity provides an adaptive advantage to secure sound sleep as well as to function correctly for daily increases in urine production in the arousal phase^5–8^. In the current study, we focused on the circadian clock function of the urothelium and presented evidence for the existence of Cx43 in the human and mouse urothelium, the daily rhythms of clock genes and Cx43 in the mouse urothelium and immortalized human urothelial cells, and the function of Cx43 as a hemichannel for ATP release. Circadian clock-regulated ATP release from Cx43 might contribute in part to the adaptation of functional bladder capacity in daily life.

An important result in this study was the finding that the bladder urothelium has a circadian rhythm generated by the circadian clock and that this indeed modulates urothelial ATP release mechanically stimulated by bladder distension in a circadian manner. Diurnal variations in ATP play a crucial role in various organisms. In rodents, the diurnal change in extracellular ATP levels in astrocytes may provide a mechanism for the clock control of gliotransmission between astrocytes and neurons, which might be associated with sleep-wake changes in the brain by modulating energy metabolism and glial activity^43,44^. Clinically, the diurnal enhancement of pain hypersensitivity might be mediated by the clock gene controlled glucocorticoid-induced enhancement of extracellular ATP release in the spinal cord^45^. As above, diurnal variations in ATP levels might be an important mechanism in nature and it is noteworthy that the physiological role of the circadian clock in the urothelium has been demonstrated here.

The mechanism of circadian ATP release from the urothelium was also demonstrated in this study. Cx43 expression had a circadian rhythm in the bladder urothelium and Cx43 functioned as a hemichannel to release ATP in urothelial cells. In addition, the concentration of mechanically induced ATP release had oscillations that correlated with Cx43 expression in the urothelium. It is generally understood that urothelial cells sense the extension stimulus and release ATP, which is essential for the micturition reflex^19,46^. Stretch-induced urothelial ATP release evokes afferent nerves within the suburothelial tissues via P2X and P2Y receptors and conveys a sense of bladder filling to the central nervous system^23–27^. Considering the diurnal change of mechanically induced ATP release via Cx43 hemichannels in the present study and the increased functional bladder capacity in *Cx43* hetero-knockout mice^11,47,48^, we infer that the diurnal rhythm of micturition is regulated by diurnal changes in the level of perception for bladder distention.

Our study had some limitations. It was unclear how and to what extent the Cx43 hemichannels are involved in the pathway of ATP release. ATP is abundant in the cell cytoplasm and is released extracellularly by several mechanisms^49,50^. Our previous study of whole bladder microarray analysis demonstrated that other genes involved in the major pathways of ATP release including Panx1 channels did not show significant circadian change^11^ (microarray data deposited in the Gene Expression Omnibus under accession code GSE35795). In addition, *Panx1*^+/+^ and *Panx1*^−/−^ mice displayed a similar phenotype in voiding micturition pattern^51^. However, it was reported that clock genes regulate the circadian expression of several genes associated with ATP release in *ex vivo* mouse bladder mucosa^52^. Although our current results do not exclude the role of other genes in the circadian change of ATP release in the urothelium because the expression amount was also regulated at multiple levels by transcriptional, post-transcriptional and post-translational mechanisms^53^, the present study demonstrated diurnal ATP release in the urothelium as well as functionally associated diurnal variation in Cx43 expression.

Another limitation is that roles of the circadian clock and ATP in physiological or pathological conditions in humans have not been investigated yet. Urothelial ATP signalling is involved in pathological conditions such as overactive bladder, but the role of P2X_2/3_ receptors is still controversial in physiological conditions^19,54,55^. Interestingly, it was suggested that the circadian clock in the bladder is modulated by endogenous purinergic receptors and that dysregulation of the interaction between the clock and major endogenous receptors may have an effect on maintaining bladder functions^56^. Further studies are expected to elucidate the roles of the circadian clock and ATP signalling in human micturition.

Despite these limitations, our study revealed that the circadian clock in the urothelium contributes to the formation of diurnal changes of ATP release through the coordination of Cx43 expression, which may modulate day–night changes in functional bladder capacity. This novel concept has the potential to lead to a new treatment strategy for nocturnal enuresis in childhood and/or frequent nocturia in the elderly, which are detrimental to the quality of life.

In conclusion, a functional circadian rhythm exists in the urothelium, and coordinates Cx43 expression and function as hemichannels that provide a direct pathway of ATP release for mechanotransduction and signalling in the urothelium. This understanding warrants further investigation for diseases characterized as disorders of micturition rhythm including nocturnal enuresis and nocturia.

## Methods

### Animals

Five-week-old female C57BL/6 mice were purchased from CLEA Japan (Tokyo, Japan) or Japan SLC, Inc. (Hamamatsu, Japan). Global *Bmal1*-knockout C57BL/6 mice were generated by mating *Probasin*-Cre; *Bmal1*^flx/flx^ mice^57–59^. Mice were housed at a constant room temperature with a cycle of 14 hours light (7:00 am to 9:00 pm) and 10 hours dark (9:00 pm to 7:00 am). Food and water were available *ad libitum*. All animal experiments were approved by the Kyoto University animal experiment committees (Permit Number: Medkyo15504), and all animals used in this study were treated according to the guidelines for animal experimentation of the experimental animal center of Kyoto University.

### Immunohistochemistry of normal human and mouse bladder

Normal human bladder tissue was obtained from a 67-year-old female patient who underwent anterior pelvic exenteration under the diagnosis of urethral malignant melanoma after informed consent. Immunostaining with Cx43 antibody (C6219, Sigma-Aldrich, St. Louis, MO, USA) was performed with normal testis as a positive control. Normal mouse bladder tissue was obtained from ten-week-old female C57BL/6 mice. Immunostaining with Cx43 antibody (C6219, Sigma-Aldrich) was performed with normal heart as a positive control. All human experiments were performed in accordance with guidelines on the use of human materials in Kyoto University, and all human experiments were approved by the ethical committees at Kyoto University Hospital. All immunostaining was performed at the Center for Anatomical, Pathological and Forensic Medical Researches in Kyoto University.

### Reverse transcriptional PCR of normal human bladder

Normal human urothelium was obtained from a 39-year-old male patient who underwent pelvic exenteration under the diagnosis of advanced rectal cancer after informed consent. Normal urothelium was obtained by scraping the bladder mucosa with a scalpel, and the suburothelium was obtained by resection with scissors. Normal human prostate was obtained as a positive control from a 73-year old male patient who underwent holmium laser enucleation of the prostate (HoLEP) under the diagnosis of benign prostatic hyperplasia. Total RNA was extracted using an RNeasy Mini kits (Qiagen, Hilden, Germany) according to the manufacturer’s protocols and complementary DNA was synthesized from 1 µg of RNA using ReverTra Ace qPCR RT Kit (TOYOBO, Osaka, Japan). The primers for PCR are shown in Supplementary Table S1. Reaction mixtures were denatured at 94 °C for 2 min, followed by 35 PCR cycles using Mastercycler nexus GSX1 (Eppendorf, Germany). Each cycle consisted of the following steps: 94 °C for 20 s, 58 °C for 30 s, and 72 °C for 35 s.

### Bioluminescence recording

The urothelium were obtained from adult *Period2*^*Luciferase*^ knock-in (*Per2::luc*) mice (Jackson Laboratories)^60^. The urothelial layer was dissected away from the smooth muscle layer using ophthalmology scissors and cultured with 800 µl of Dulbecco’s Modified Eagle Medium (DMEM, Sigma-Aldrich), supplemented with 10 mM HEPES (pH 7.2), 2% B27 (Thermo Scientific, Rockford, IL, USA), 25 units/ml penicillin, 25 mg/ml streptomycin, and 1 mM luciferin, in 35-mm dish, and air sealed. Bioluminescence was continuously monitored without interruption for 7 days immediately upon placement in culture with a dish-type photon countable luminometer (Kronos Dio, ATTO, Tokyo, Japan) at 35 °C.

### Tissue preparation

Eight-week-old female C57BL/6 mice anesthetized with isoflurane and euthanized by cervical dislocation, were analysed at seven consecutive time points every 4 hours during the day under a dim light (n = 6 for each time). The bladders were removed and sliced open in a 6-cm dish containing cold normal saline. We scraped the bladder mucosa with a cell scraper in cold normal saline, and collected the normal saline including desquamated urothelium. It was immediately cryopreserved in liquid nitrogen for protein assay, or mixed with RNAlater (Sigma-Aldrich) preserved at 4 °C for mRNA assay. The remaining suburothelium and smooth muscle layer were gathered together as a reference, and a part of the liver was used as a control.

### Cell culture

The hTERT-immortalized human urothelial cell line (TRT-HU1) generated by Dr. Louis S. Liou was a gift from Dr. Rosalyn M. Adam (Urological Diseases Research Center, Children’s Hospital Boston, Boston), and hTERT-immortalized normal human urothelial cells (TERT-NHUC) were generated and kindly provided by Dr. MA Knowles (Cancer Research UK Clinical Centre, St James’s University Hospital, Beckett Street, Leeds LS97TF, UK). TRT-HU1 cells were maintained in DMEM (Sigma-Aldrich) containing 2 mM L-glutamine and 110 mg/L sodium pyruvate supplemented with 15% FBS (GIBCO), non-essential amino acids (GIBCO), and 1.15 mM 1-thioglycerol, as previously described^32^. TERT-NHUC cells were maintained in KSFM (Life Technologies Ltd, Paisley, UK) supplemented with the supplied bovine pituitary extract and epidermal growth factor in a humidified atmosphere of 5% CO_2_ at 37 °C, as previously described^36^.

### Serum shock analysis of hTERT-cells

As previously reported^38^, TRT-HU1 were cultured until sub-confluent in DMEM with 15% FBS followed by 48 hours incubation in DMEM with 0.5% FBS. The cells were treated with 50% horse serum (GIBCO) in DMEM for 2 hours, and washed twice with DMEM and then maintained in DMEM with 0.5% of FBS for a maximum of 72 hours. The time 0 (hr) in the figures indicates before serum shock.

### Immunostaining of hTERT-cells

After washing with TBS (3 times, 5 min), the cells were permeabilized for 30 min with 0.25% Triton X-100 (in TBS), blocked for 60 min with 1% bovine serum albumin (BSA, in TBS) and incubated 2 days with primary antibodies (in 1% BSA + 0.4% Triton X-100) at 4 °C. The following primary antibodies were used: rabbit polyclonal Cx43 antibody (C6219, Sigma-Aldrich, 1:500), mouse monoclonal anti-vimentin antibody (ab8978, 1:100, Sigma-Aldrich). Donkey anti-mouse IgG (H + L) Alexa Fluor 488 (ab150105, Abcam, Cambridge, UK) was used as secondary antibody followed by DAPI nuclear staining.

### Real-time quantitative RT-PCR analysis

Total RNA extraction and complementary DNA synthesis from the mouse urothelium or cultured cells was done using the same procedure as for the human urothelium. Real-time quantitative RT-PCR was performed with SYBR Green PCR Master Mix (Life Technologies, Carlsbad, CA, USA) and a 7300 Real-time PCR system (Life Technologies). The thermal cycling conditions were 94 °C for 15 s, 60 °C for 15 s, and 72 °C for 1 min. Values were adjusted relative to the expression levels of the housekeeping gene *Gapdh* or 18 s ribosome. Primers used are listed in Supplementary Table S1. The ΔΔCt method was used to determine the relative gene expression of the genes of interest.

### Immunoblotting

Whole cell lysates from bladder urothelium and cultured cells were lysed with radioimmunoprecipitation assay (RIPA) buffer containing proteinase inhibitors, which were resolved by SDS-PAGE and transferred to polyvinylidene difluoride membranes (Millipore, Bedford, MA, USA) using a Mini Trans-Blot Cell system (Bio-Rad Laboratories). Membranes were blocked with 5% bovine serum albumin diluted in TBST (BSA/TBST) and incubated with primary antibodies diluted in 1% BSA/TBST followed by incubation with horseradish peroxidase-conjugated secondary antibodies diluted in 1% BSA/TBST and developed for reading by enhanced chemiluminescence (SuperSignal West Pico Chemiluminescent Substrate, Thermo). Images were acquired with the LAS-4000 imaging system (Fujifilm Life Science, Tokyo, Japan). Anti-Cx43 (C6219, Sigma-Aldrich, 1:8000), anti-BMAL1 (ab93806, Abcam, 1:500), anti-beta actin (ab6276, Abcam, 1:5000) and anti-GAPDH (GAPDH, 2118, Cell Signaling Technology, Danvers, MA, USA, 1:5000) were used as the primary antibodies.

### Cell transfection

Stable knockdown of *Cx43* or *BMAL1* was accomplished using shRNA. Lentivirus-based plasmids containing TRC2-pLKO-puro shRNA sets to human *BMAL1* (SHCLNG-NM_001178; TRCN0000331011 (sh1), TRCN0000331012 (sh2), TRCN0000331014 (sh3), TRCN0000331078 (sh4), TRCN0000331079 (sh5) and the same sets to human *Cx43* (SHCLNG-NM_000165; TRCN0000333535 (sh11), TRCN0000344841 (sh12), TRCN0000344842 (sh13), TRCN0000344843 (sh14), TRCN0000353147 (sh15)) were purchased from Sigma-Aldrich. Non-silencing shRNAs (Sigma-Aldrich) were used as negative controls. To obtain Cx43-overexpressing urothelial cells, Precision LentiORF GJA1w/Stop Codon (OHS5897-202619897) and Precision LentiORF RFP Control Glycerol Stock (OHS5832) were purchased from Open Biosystems (Huntsville, AL, USA). The experimental procedure for transfection was performed according to the Sigma-Aldrich or Open Biosystems technical manual.

### Measurement of ATP release *in vivo* and *in vitro*

After eight-week-old female C57BL/6 mice with or without *Bmal1-*knockout were anesthetized with 4% (induction) and 2% (maintenance) isoflurane, a 24 G catheter (SR-OT2419C, Terumo, Tokyo, Japan) was inserted into the urethra and clamped with a clip (AM-1, Natsume Seisakusho Co. Ltd., Tokyo, Japan), and connected to a tube filled with phosphate buffered saline (PBS). After bladder distention with 30 cm H_2_O for 10 minutes, we collected the PBS from the bladder lumen. The ATP concentration in the samples was calculated from standard curves constructed using ATP from 20 nM to 20 μM.

### Measurement of ATP release *in vitro*

The luciferin-luciferase assay (CellTiter-Glo^®^2.0 Assay; Promega, Madison, WI, USA) was used to quantify the amounts of ATP released in the bathing solution of cultured cells in response to rinsing-induced mechanical stimulation^27^. Briefly, 20 μl of bathing solution samples were individually placed in triplicate in white walled 96-well plates (Nunc F96 MicroWell; Thermo), and 100 μl CellTiter-Glo^®^2.0 reagent was added directly to each well. Plates were incubated at room temperature for 15 minutes and were transferred to the Multilabel Plate Reader VICTOR X5 (PerkinElmer, Waltham, MA, USA), where luminescence was measured using a 1-sec integration time. The ATP concentration in the samples was calculated with the same procedure as for the *in vivo* study.

### Dye-transfer experiment

Dye-transfer experiment was performed with Lucifer yellow (LO259, Sigma). In 6 cm dishes, confluently cultured cells were cut with a diamond cutter in the presence of Lucifer yellow followed by 4% PFA fixation. Images in fluorescence along with the cutting line were taken and the dye-transfer lengths from the cutting edge were measured using ImageJ software (http://rsb.info.nih.gov/ij/).

### Reagents

Carbenoxolone disodium salt (CBX), GAP19 peptide (KQIEIKKFK) and GAP19 scramble peptide (IKFKKEIKQ) were purchased from Sigma-Aldrich.

### Statistical analysis

All data are expressed as the mean ± s.e.m. JMP Pro 11 (SAS Institute Inc., Cary, NC, USA) was used for statistical analysis. Unpaired t-test, one-way ANOVA with Dunnett’s *post hoc* test, one-way ANOVA with Tukey’s *post hoc* test were performed when appropriate. The *P*-values of gene oscillation were calculated using a free Cosinor analysis software (Version 3.1) available at http://www.circadian.org/softwar.html. *P* < 0.05 was regarded as statistically significant. In the figures, statistical significance is indicated as follows: *P < 0.05, **P < 0.01, and ***P < 0.001.

## Electronic supplementary material

Supplementary information
