# Supplementary material for: Circadian coordination of ATP release in the urothelium via connexin43 hemichannels
Source: Sci Rep. 2018 Jan 31;8:1996. doi: 10.1038/s41598-018-20379-0 (PMC5792455; doi:10.1038/s41598-018-20379-0)
Supplement: Supplementary file 1 — Supplementary information [file 41598_2018_20379_MOESM1_ESM.pdf]

Supplementary Information for

Circadian coordination of ATP release in the urothelium via connexin43 hemichannels

Atsushi Sengiku[1], Masakatsu Ueda[1], Jin Kono[1], Takeshi Sano[1], Nobuyuki Nishikawa[2], Sumihiro Kunisue[3], Kojiro Tsujihana[3,4], Louis S. Liou[5], Akihiro Kanematsu[6], Shigeki Shimba[7], Masao Doi[3], Hitoshi Okamura[3] ,Osamu Ogawa[1] and Hiromitsu Negoro[1*]

[1]Department of Urology, Graduate School of Medicine, Kyoto University, Kyoto, 606-8507, Japan
[2]Department of Urology, Japanese Red Cross Otsu Hospital, Shiga, 520-8511, Japan
[3]Department of Systems Biology, Graduate School of Pharmaceutical Sciences, Kyoto University, Kyoto, 606-8501, Japan
[4]Department of Dermatology, Graduate School of Medicine, Kyoto University, Kyoto, 606-8507, Japan
[5]Department of Urology, Cambridge Health Alliance, MA, 02139, USA
[6]Department of Urology, Hyogo College of Medicine, Hyogo, 663-8501, Japan
[7]Department of Health Science, School of Pharmacy, Nihon University, Chiba, 245-8555, Japan

Correspondence should be addressed to
H.N (E-mail: hnegoro@kuhp.kyoto-u.ac.jp).

Supplementary information includes:

Figures S1 to S13
Table S1

Supplementary Figure S1

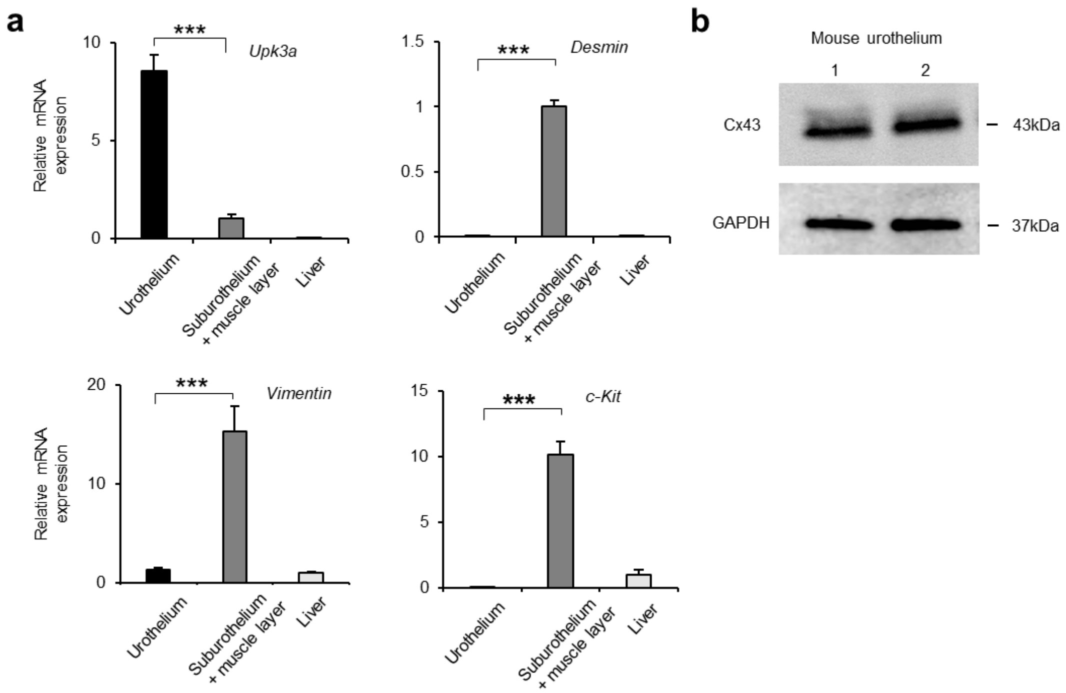

Supplementary Figure S1. Normal mouse urothelium expresses Cx43. (**a**) The collection accuracy of the urothelium using real-time quantitative RT-PCR analysis with specific markers for each layer of the bladder; Uroplakin 3a (Upk3a) for the urothelium, vimentin and c-kit for the suburothelium, and desmin for the muscle layer. Markers other than Upk3a were significantly low in the urothelium indicating that the sample of urothelium was collected selectively. ***P<0.001 by Student's t-test (n=3). Error bars indicate s.e.m. (**b**) Immunoblotting analysis shows Cx43 expression in mouse urothelium. Each sample was obtained from a different mouse.

Supplementary Figure S2

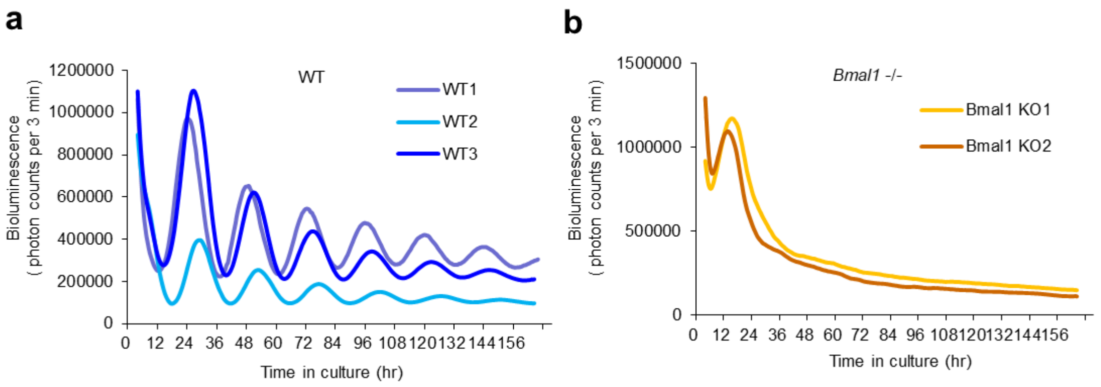

Supplementary Figure S2. Disturbed oscillations of the urothelial internal clock in *Bmal1* KO; *Per2::luc* mice. (**a**) Oscillation of bioluminescence in urothelium obtained from *Per2::luc* mice in an *ex vivo* culture. The period of oscillation was 24.13±0.07 (mean±s.e.m.) (n=3). The age and sex of each mouse is as follows: WT1: five-month-old, female, WT2: five-month-old, male, and WT3: seven-week-old, female. (**b**) The oscillation of bioluminescence in *Bmal1 KO*; *Per2::luc* mice was lost completely (n=2). The age and sex of each mouse is as follows: *Bmal1* KO1: six-month-old, male, and *Bmal1* KO2: seven-week-old, female.

Supplementary Figure S3

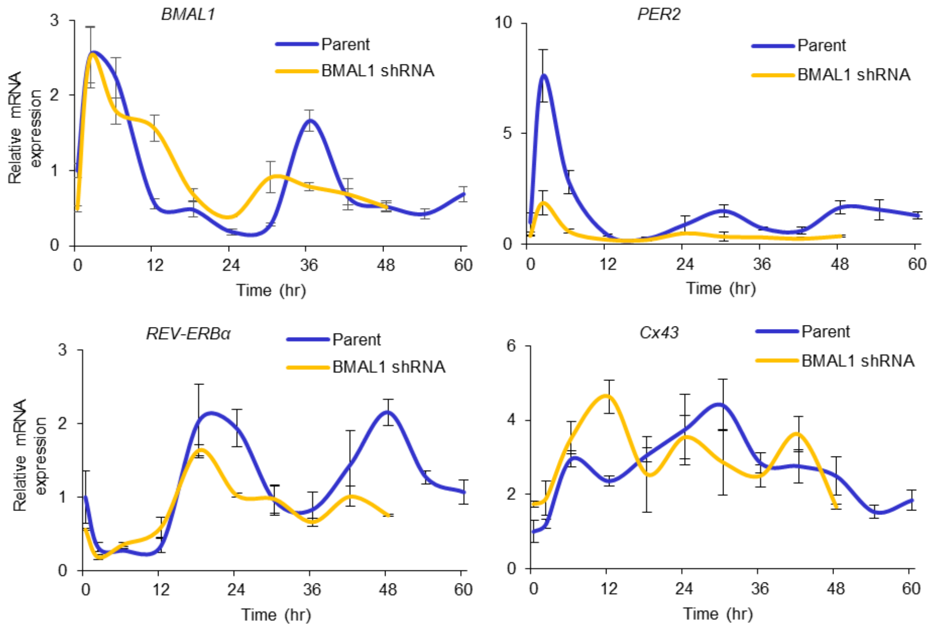

Supplementary Figure S3. Disturbed oscillations of clock genes and *Cx43* mRNA accumulation in *BMAL1* shRNA transfected TRT-HU1. Merged temporal variation in mRNA accumulation of clock genes and *Cx43* mRNA levels in serum-shocked parent TRT-HU1 and *BMAL1* shRNA TRT-HU1 (sh2 TRT-HU1). Circadian rhythms seen in the parent TRT-HU1 had almost disappeared in sh2 TRT-HU1. Error bars indicate s.e.m.

Supplementary Figure S4

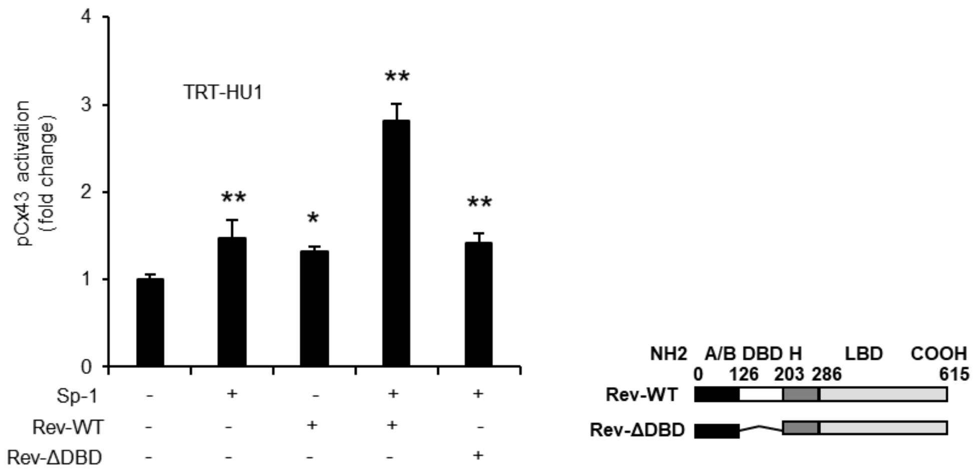

Supplementary Figure S4. Promoter-reporter assay in human urothelial cells. Rev-erbα and Sp-1 upregulated the transcription of *Cx43* mRNA in TRT-HU1 cells. *P<0.05 and **P<0.01 vs. control (all none) by Student's *t*-test (n=3). All error bars indicate s.e.m. DBD, DNA-binding domain.

<u>Supplementary Figure S5</u>

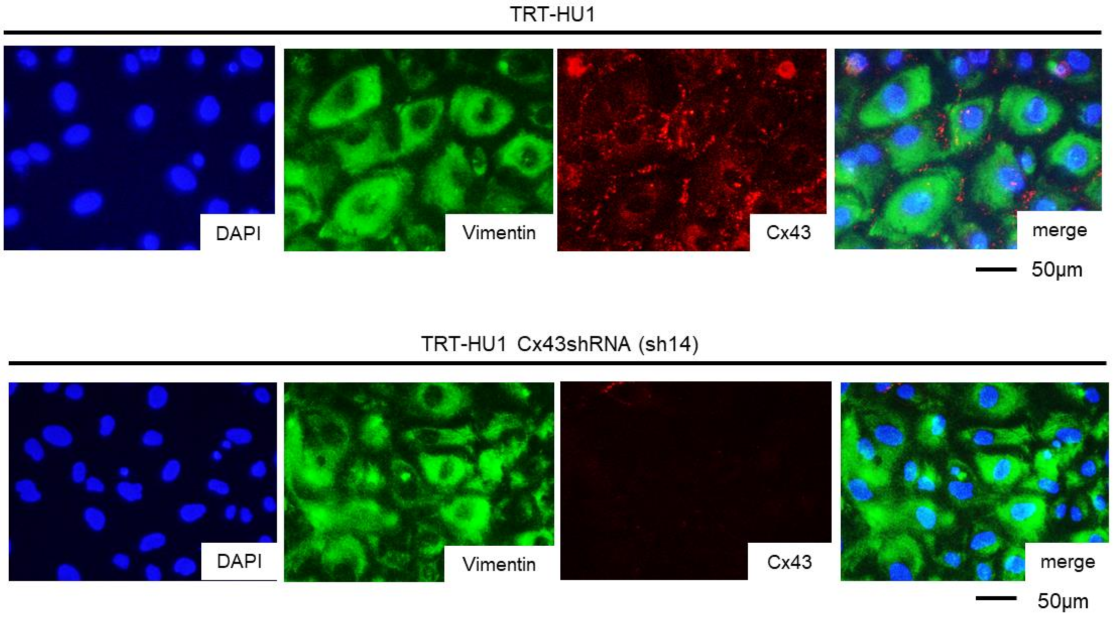

Supplementary Figure S5. Immunostaining of Cx43 in TRT-HU1 and *Cx43* shRNA transfected TRT-HU1. *Cx43* shRNA TRT-HU1 (sh14 TRT-HU1) showed less Cx43 expression than the parent TRT-HU1.

Supplementary Figure S6

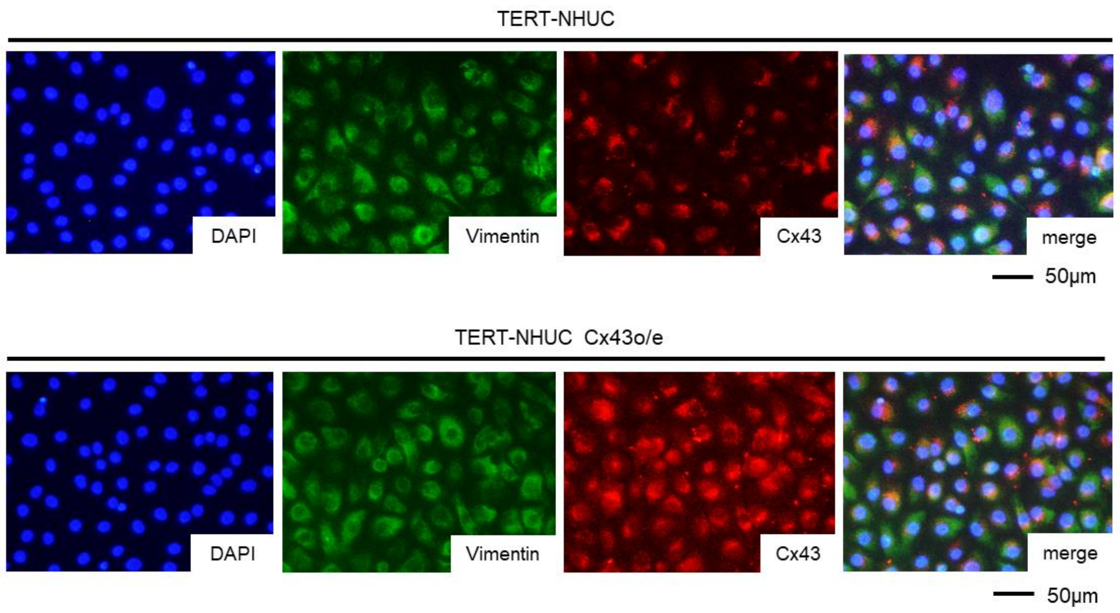

Supplementary Figure S6. Immunostaining of Cx43 in TERT-NHUC and *Cx43*-overexpressing TERT-NHUC. *Cx43*-overexpressing TERT-NHUC showed higher Cx43 expression than the parent TERT-NHUC.

Supplementary Figure S7

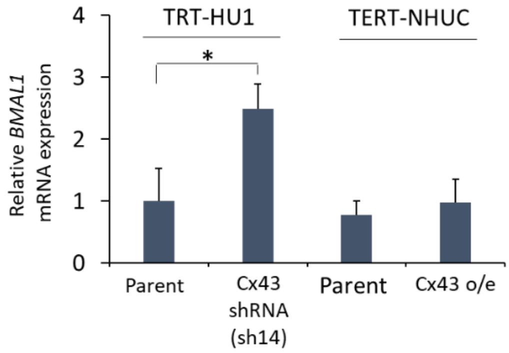

Supplementary Figure S7. Relative *BMAL1* mRNA expression level in *Cx43* knockdown/overexpressing human urothelial cells. *BMAL1* mRNA expression level was significantly increased in *Cx43* knockdown cells (n=3, *P<0.05 by Student's t-test) and was static in *Cx43* overexpressing cells (n=3). These results suggest that the ATP release from urothelial cells is under more direct control of Cx43 rather than that of Bmal1 or other possible genes for activation of ATP release driven by *Bmal1* if existed. All error bars indicate s.e.m.

Supplementary Figure S8

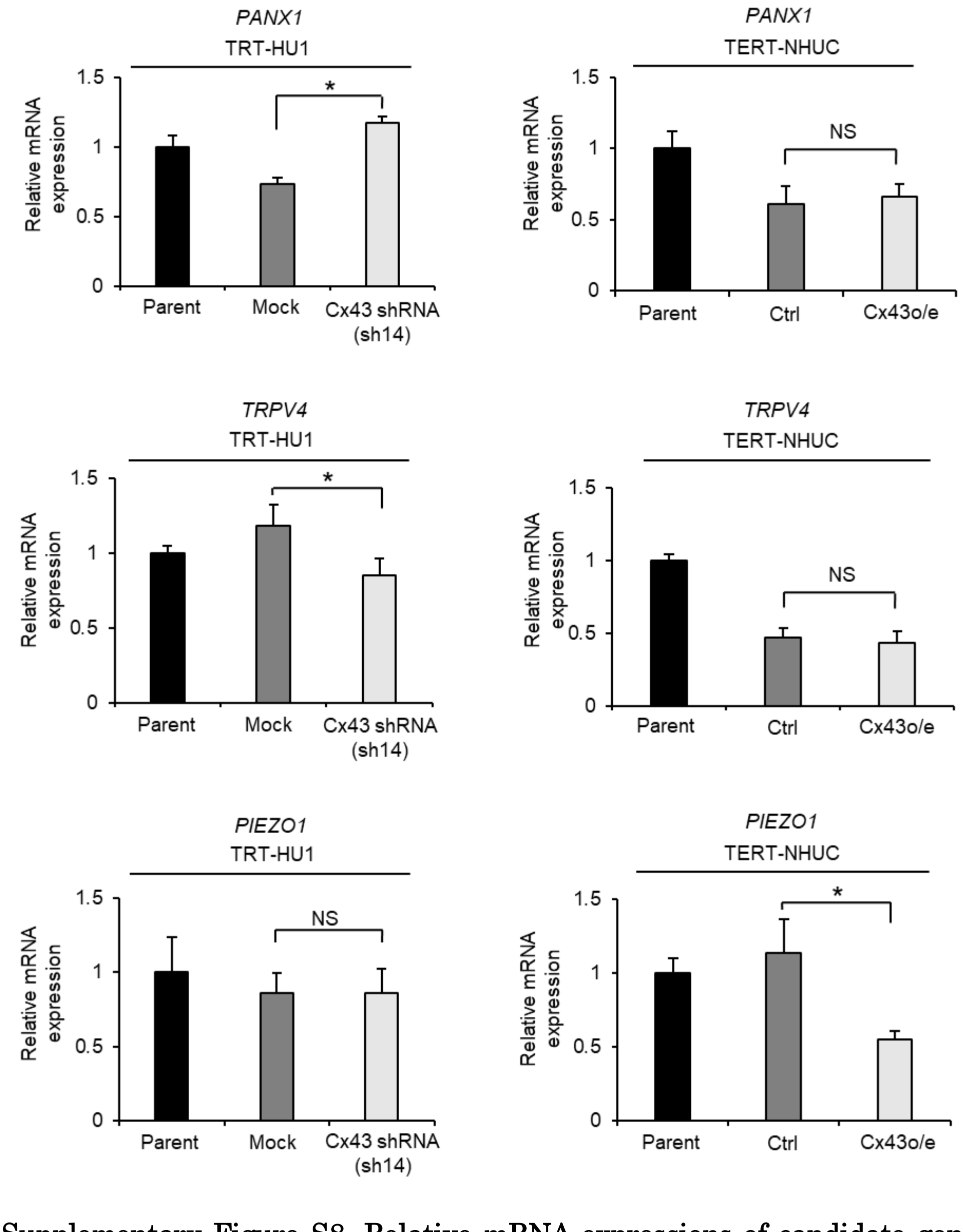

Supplementary Figure S8. Relative mRNA expressions of candidate genes associated with urothelial ATP release, *PANX1, TRPV4, PIEZO1* and *VNUT* in *Cx43* knockdown/overexpressing cells. *PANX1* mRNA expression was increased and *TRPV4* mRNA expression was decreased in *Cx43* knockdown TRT-HU1 cells. These mRNA expressions had no significant changes in *Cx43* overexpressing TERT-NHUC cells. *PIEZO1* mRNA expression did not significantly change in the *Cx43* knockdown cells, and was significantly decreased in the *Cx43* overexpressing cells. *VNUT* mRNA was not detected in two immortalized human urothelial cells and commercially available human

urothelial cells (#4320, ScienCell Research Laboratories, Carlsbad, CA, USA). All sample size is n=3, *P<0.05 by Student's t-test, and all error bars indicate s.e.m. NS, not significant P-value; o/e, overexpression.

Supplementary Figure S9

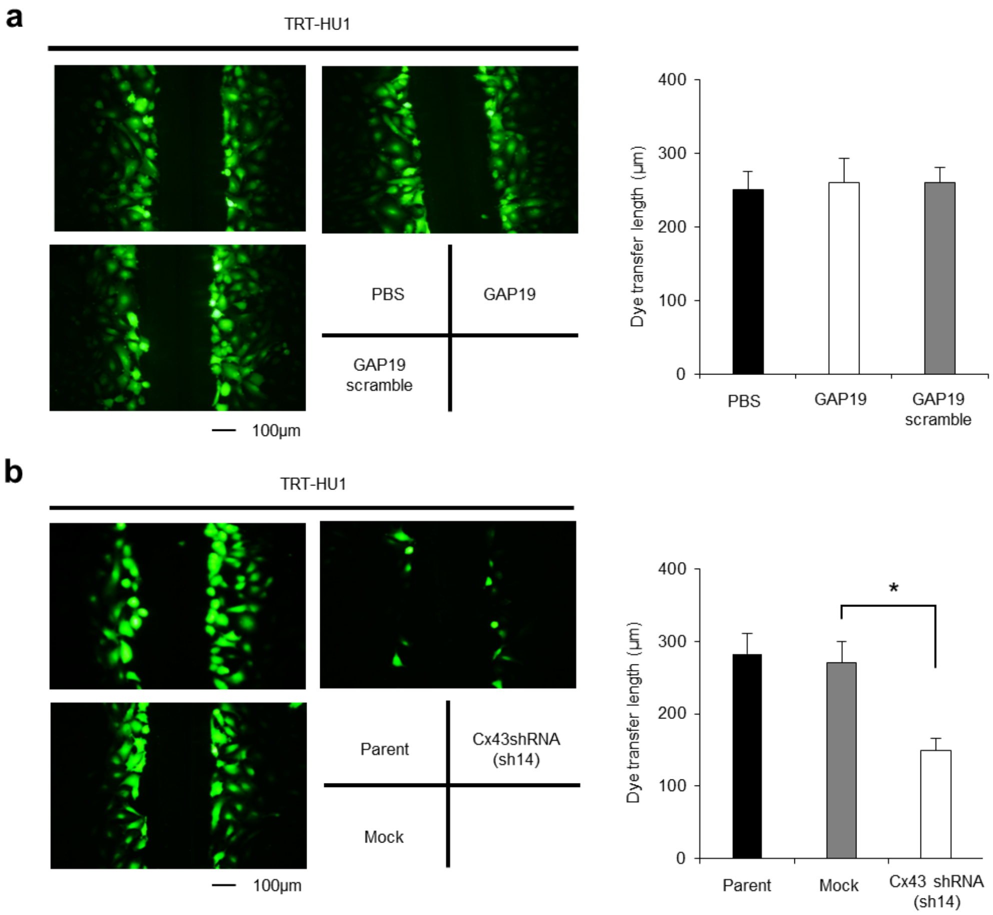

Supplementary Figure S9. Dye-transfer experiment. (**a**) GAP19 and GAP19 scramble peptide did not prevent dye transfer. The average dye-transfer length was shown as bar chart (n=12, mean±s.e.m.). (**b**) Dye-transfer lengths were decreased in Cx43 shRNA cells (sh14 TRT-HU1) significantly (n=12, *P<0.05 by Student's t-test, mean ± s.e.m.).

<u>Supplementary Figure S10</u>

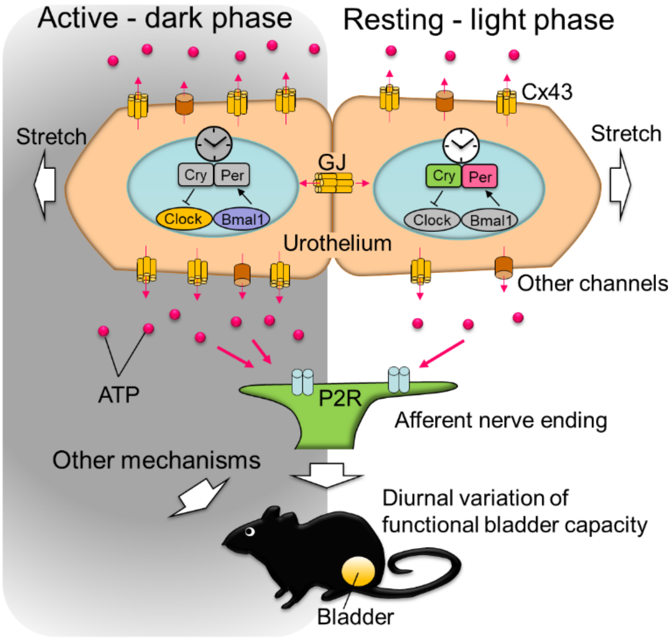

Supplementary Figure S10. A schematic model illustrating circadian rhythm in the urothelium coordinates ATP release via Cx43 hemichannels to modulate functional bladder capacity. The circadian clock exists in the urothelium, which coordinates diurnal changes of Cx43 expression and function as hemichannels for ATP release. Diurnal changes of ATP release during the bladder distension may lead to diurnal variation of bladder afferent activity presumably via the activation of P2 purinergic receptors (P2R) on the afferent nerve ending to modulate functional bladder capacity with other mechanisms. GJ, gap junction.

Supplementary Figure S11

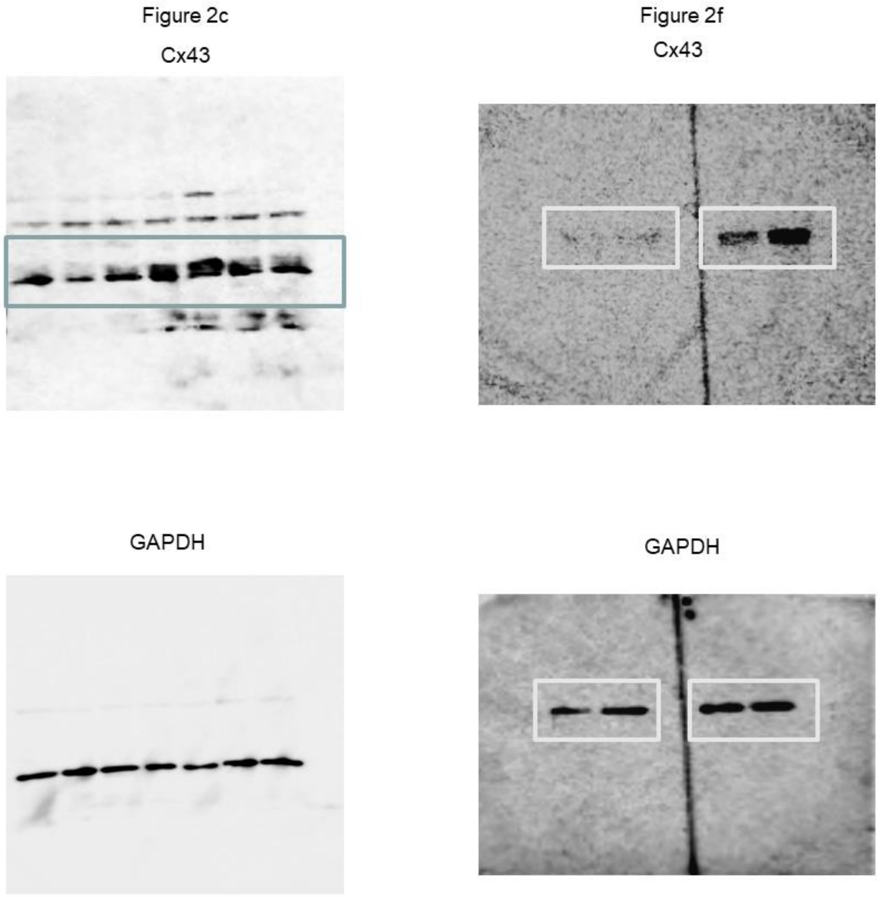

Supplementary Figure S11. Full-length unedited blots for Figures 2c and 2f.

Supplementary Figure S12

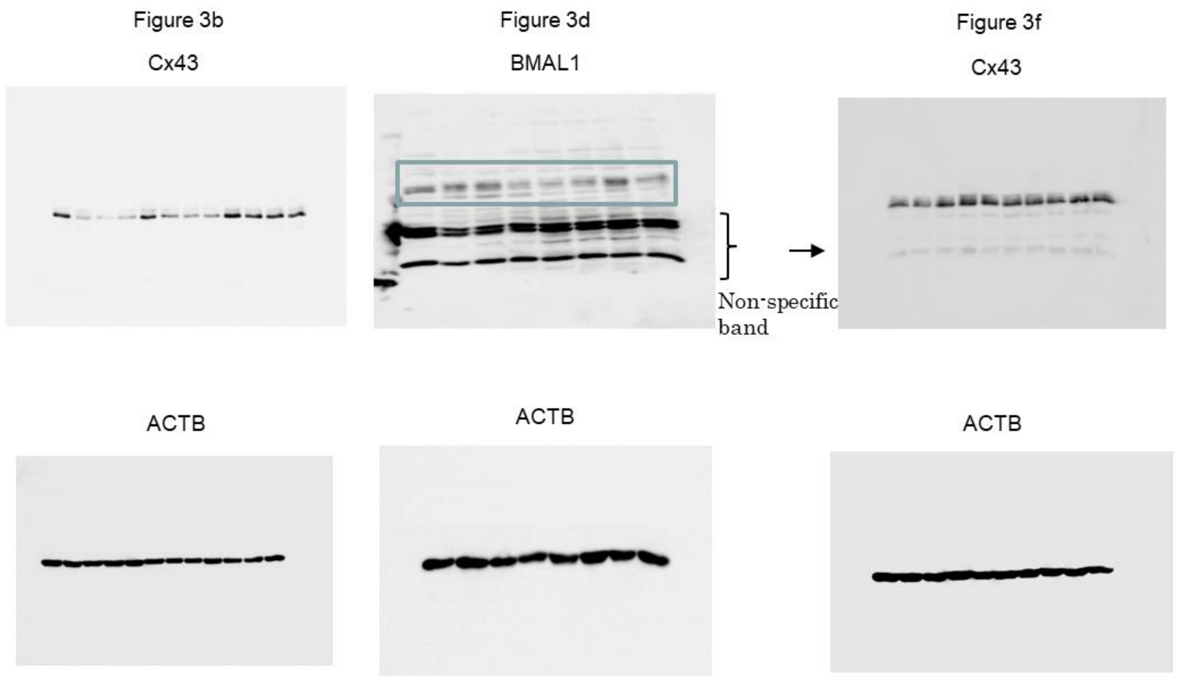

Supplementary Figure S12. Full-length unedited blots for Figures 3b, 3d and 3f.

Supplementary Figure S13

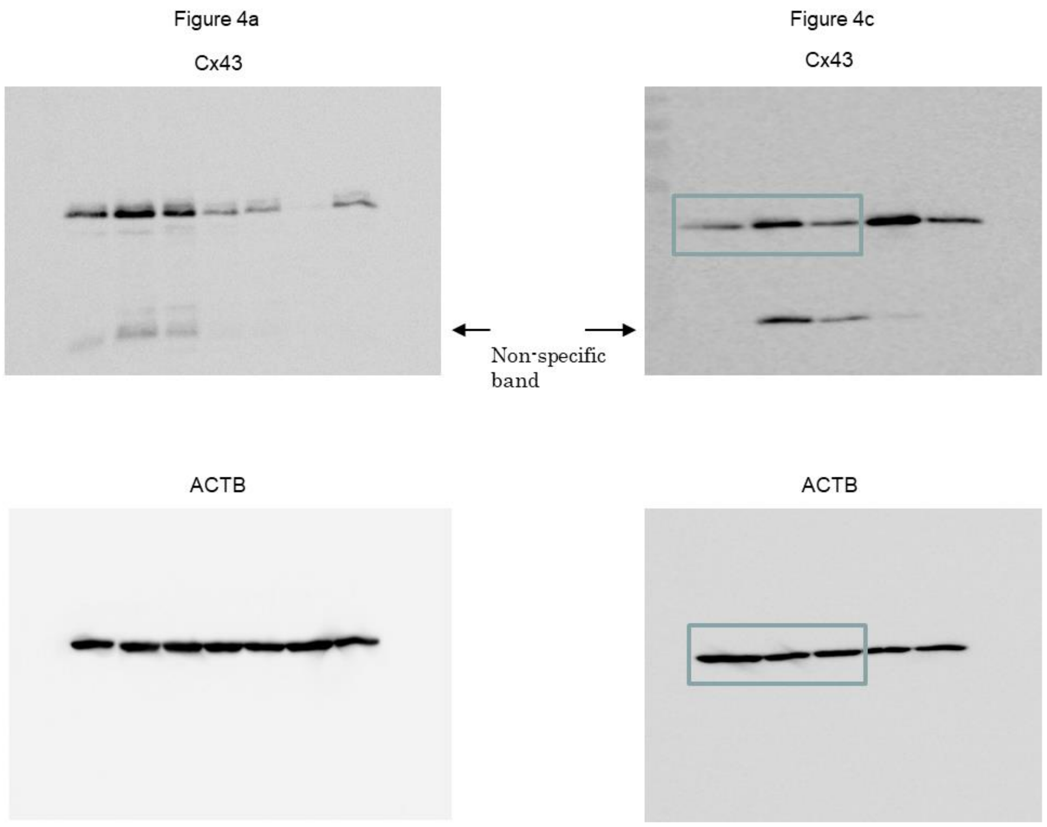

Supplementary Figure S13. Full-length unedited blots for Figures 4a and 4c.

Supplementary Table S1

Species	Gene name		Primer sequences	Amplicon size (b.p.)
Human	*Cx43*	Forward	AGCAAAAGAGTGGTGCCCA	63
		Reverse	TTGTCAAGGAGTTTGCCTAAGG	
	BMAL1	Forward	GAAGACAACGAACCAGACAATGAG	86
		Reverse	GGTTGTGGAACTACATGAGAATGC	
	PER2	Forward	TGGACTCCTCGGCTTGAAAC	179
		Reverse	GGAACGAAGCTTTCGGACCT	
	REV-ERB α	Forward	GTTTGCCAAACACATCCCG	101
		Reverse	AAGCAAAGCGCACCATCAG	
	CK20	Forward	CAAAAAGGAGCATCAGGAGGA	63
		Reverse	CACATTGACAGTGTTGCCCAG	
	DES	Forward	GGACCTGCTCAACGTGAAGA	132
		Reverse	GGGCTGGTTTCTCGGAAGTT	
	GAPDH	Forward	GAAGGTGAAGGTCGGAGTC	226
		Reverse	GAAGATGGTGATGGGATTTC	
	PANX1	Forward	GGCAGAGCTCCAAGGTATGAA	186
		Reverse	GCAAACCAGCTGTGAAACCA	
	TRPV4	Forward	AGCAAGATTGAGAACCGCCA	133
		Reverse	AGATGACCATGGCACACAGG	
	PIEZO1	Forward	GACCCTCTCGCGACACATAG	178
		Reverse	ATCATCATCCAGCTCCCGTG	
	VNUT	Forward	TGTGTATGCATGACCCTGAC	259
		Reverse	CACCCCCAATCCGATCCC	
Mouse	*Upk3a*	Forward	CATCATCCTCAGCTTTGTGGA	159
		Reverse	GAGAACACCTCTGCTCTGTCTAGG	
	Des	Forward	AAGGCCAAACTACAGGAGGAAA	97
		Reverse	TACGAGCTAGAGTGGCTGCATC	
	Vim	Forward	AAGTCCAAGTTTGCTGACCTCTC	93
		Reverse	CTGTCTCCGGTACTCGTTTGAC	
	c-Kit	Forward	CCAACTTCGCCTGACCAGAT	429
		Reverse	GCCTGGATTTGCTCTTTGTTGT	
	Cx43	Forward	CCATCCAAAGACTGCGGAT	138
		Reverse	GTAATTGCGGCAGGAGGAA	
	Bmal1	Forward	CCAAGAAAGTATGGACACAGACAAA	80
		Reverse	GCATTCTTGATCCTTCCTTGGT	
	Per2	Forward	CACACTTGCCTCCGAAATAACTC	79
		Reverse	AGCGCACGGCTGTCTGA	
	Rev-erb α	Forward	ACAGCAGCCGAGTGTCCC	70
		Reverse	ACACAGTAGCACCATGCCATTC	
	Gapdh	Forward	GCACAGTCAAGGCCGAGAAT	151
		Reverse	GCCTTCTCCATGGTGGTGAA	
	Rn18S	Forward	ACTCAACACGGGAAACCTCA	123
		Reverse	AACCAGACAAATCGCTCCAC	

Supplementary Table S1. Primers for real-time quantitative RT-PCR. Primers were designed using Primer-BLAST or according to previous reports[11,12].

Supplementary Methods

Promoter–reporter assay

Reporter plasmids with various expression vectors were transfected into TRT-HU1 cells using NEPA21 electroporator (Nepa Gene Co. Ltd., Japan). According to the manufacturer's protocol, pCx43-luc 1722 ng and pTK-RL 278 ng were transfected with various amounts of expression vectors (total 10 μg) to 1×10^6 TRT-HU1 cells. Based on the introduction efficacy of pCMV-EGFP, the optimum condition of electroporation was determined as below: poring pulse was 150 V, range 2.5 ms, interval 50 ms, twice, attenuation rate 10%, polar character +, and transfer pulse was 20 V, range 50 ms, interval 50 ms, five-times, attenuation rate 40%, polar character +/-. Plasmid dosage was kept constant by pCMV-EGFP vector. Lysates were harvested 48 hr posttransfection in 6-well plate (IWAKI, Japan), and the luciferase activity was measured using a dual luciferase assay reagent (Promega).
